# Agnes Kalibata: transforming food systems

**DOI:** 10.2471/BLT.21.031021

**Published:** 2021-10-01

## Abstract

Agnes Kalibata talks to Gary Humphreys about the need for multisectoral food system reform.

*Q: You were raised as a refugee in Uganda. How did your early experiences influence your life and career?*

A: I was born in Rwanda to parents who were displaced during Rwanda’s struggle for independence in the early 1960s. That was how I came to be raised in a refugee camp, where my parents grew beans and maize, and kept cows. The experience helped me appreciate several things, like how critical milk is to the growth of young children! Families would literally work to be paid with milk. I also understood how important culture is in the way we produce and consume food. For example, my parents made us throw away fish because eating fish was “not what we did”. Perhaps most important, I came to appreciate that farming can be more than a means of subsistence and that, by generating income, can drive prosperity with all the implications that has for health and well-being. It seems to me that I have spent most of my career encouraging smallholders (small-scale farmers) to embrace farming as a way out of poverty, helping them combat pests, and improving their access to the seeds, livestock and markets they need. I say smallholders, but it is important to remember that in the African context, smallholders are also big in the sense that around 80% of agricultural production in Africa comes from them.

*Q: You studied entomology, at bachelor and doctoral level. How has that fed into your work on agriculture and nutrition?*

A: My education was largely focused on understanding the management of pests and diseases affecting agricultural systems. This is a huge issue in the African context and with climate change is likely to become bigger, as evidenced by the recent increase in desert locust swarms that have been impacting East African nations since the beginning of 2020. Effective management of pests and diseases is key to achieving optimal agricultural yields and ensuring food security. When I took up the position of Rwandan Minister of Agriculture in 2008, I set a target of raising average calorie levels from 1700 kcal daily to the recommended 2100 kcal per day, and to shift from a very low protein base to help tackle the country’s high levels of malnutrition and stunting. All hands were on deck at that time and I got the support I needed to meet those goals. My entry into government also happened to coincide with the One Cow per Poor Family programme launched by the president in 2006, and which I helped implement.

“Ending hunger is going to require ambitious action at scale.”

*Q: What insights did you gain in moving from academia into the political arena?*

A: My research had always been applied (seeking to solve practical problems) and conducted within farm settings, so the transition to policy was relatively easy. What was really eye-opening to me was how long it takes to translate scientific evidence into implementable policy. I have come to appreciate that this is not a problem unique to Rwanda, but can be observed in many African countries where it can take as much as 10 years to translate knowledge into policy. This is unfortunate, because smallholders need science-based policy and guidance to achieve better returns on labour and strengthen their resilience while ensuring sustainable use of public goods such as clean water and air.

*Q: Can you give some examples of how science-based policy can help small farmers?*

A: A good example is policy encouraging or supporting the use of drought-resistant or higher yield crop varieties. These are critical to increasing the resilience of family farms in the face of climate change, while also allowing them to generate the surplus required to support a viable business. Better yielding varieties have enabled smallholders in Africa to increase production from around 0.5 metric tonnes of maize per year to about five metric tonnes, currently. At that level of production, farmers are not only feeding their families and connected communities – they become self-sufficient and can send their children to school.

Supporting the judicious use of fertilizers is another example. Fertilizers have played a critical role in feeding people and ending hunger and poverty around the world and are of great importance in Africa where soils can be heavily depleted and can lack key nutrients. Appropriate fertilizer blends with nutrients like calcium and zinc have been proven to reduce malnutrition by improving the nutritional quality of grain and can impact the health of millions of people at relatively low cost. That said, given the current evidence on environmental impact of overuse and misuse of fertilizers, it is clear that we need to invest in innovations that reduce the amount of fertilizers used. This does not mean that fertilizers should be abandoned. In fact, some experts have suggested that African food systems and ecosystem services will deteriorate further without increased and judicious use of fertilizers, because farmers will keep opening up new land encroaching on forests, wetlands and protected areas to feed a growing population. Farmland lost to agricultural degradation currently constitutes around 60% of the total land farmed in Africa and this is not sustainable. So, going forward we need to consider innovations that have the potential to contribute to sustainability.

It is also important to note that innovations introduced on the farm have knock-on effects in the food system downstream. For example, the introduction of high-yield, drought-resistant crops benefits not just the farmer but also the small and medium-sized enterprises with whom the farmer trades, and who ensure farmers’ products come to market. In other words, the benefits are felt throughout the food system and not just by the food producer. It is important that we consider such cross-sectoral impacts in order to make progress.

Indeed, cross-sectoral collaboration is vital not only to reducing hunger and malnutrition, but also to minimizing the negative impact of food systems on the environment, and to ensure prosperity and support development. Such collaboration was something I experienced from the very beginning in my time as Rwanda’s agriculture minister, with different ministries coming together to resolve issues quickly, and I somewhat took it for granted. Now, working across Africa as president of AGRA, I see that traditional silos tend to prevail.

*Q: Can you say more about that?*

A: I am talking about the tendency for people to look at challenges from their particular perspective or expertise – including, of course, health – failing to recognize that food or nutrition impacts and is impacted by many different sectors. The SDGs (sustainable development goals) reflect the intersectoral nature of the challenges we face as do the most helpful initiatives in the field. The Rwandan ‘One Cow per Poor Family’ programme is a good example. The cow produces milk that meets certain nutrition needs, while the manure generated by the cow is put into a small biogas digester where the methane is removed and used for cooking before being applied to the plot where farmers can grow vegetables that feed their families or generate income. Because there is no need to cut trees for fuel the impact on the environment is reduced, while burning methane instead of biomass reduces indoor and ambient air pollution. This is crucial for advancing the public health agenda, while helping reduce methane emissions – a key climate goal. The programme is a whole-of-government endeavour – involving the ministries of agriculture, health and energy – and for me exemplifies the systems approach to family farm food production.

“There is broad consensus regarding the need for change.”

We need to support the development of such projects and seek out examples where they exist and learn from them, recognizing that there are many innovative solutions available that, with the right support, can transform food systems to simultaneously improve the health of people, animals and the planet. UN agencies are already aligning to support such initiatives, examples including the WHO (World Health Organization) and UNICEF (United Nations Children’s Fund) Global Coalition for Children’s Diets, the Tripartite Alliance on One Health, and the Committee on World Food Security Voluntary Guidelines on Food Systems and Nutrition, but more can be done. The need to come together, embracing a systems perspective, was one of the key messages that came out of the UN Food Systems Summit, held last month, with the aim of maximizing the co-benefits of a food systems approach across the entire 2030 SDG Agenda while also helping meet the challenges of climate change. Key to achieving those aims is to facilitate the sharing of ideas and lessons learnt, which was the main purpose of the summit’s five Action Tracks which offer stakeholders from a wide range of backgrounds a space to share and learn, with a view to fostering new actions and partnerships and amplifying existing projects. The Action Tracks helped bring together and tap into some promising ideas from all over the world, and these have been consolidated into 15 action areas.

*Q: According to a recent multi-agency UN report, there was a dramatic worsening of world hunger in 2020, in part due to the ongoing pandemic, which puts in doubt the SDG commitment to ending hunger by 2030. What can be done to get us back on track?*

A: Ending hunger is going to require ambitious action at scale, supported by increased investment and innovation. It will also require sharing the costs of trade-offs such as those between agricultural production and environmental sustainability, and the reduction of waste in all forms. Most of all, it will require the collective will and engagement of billions, not millions of people. Food systems governance will be key, especially in the implementation of multisectoral approaches. Governance is also critical to bringing in people from outside the public sector including private sector stakeholders, civil society, indigenous peoples, youth and others to ensure that their voices are heard, and their participation guaranteed.

As daunting as all that may seem, my experience as special envoy to the UN summit convinced me that there is broad consensus regarding the need for change. That represents a tremendous opportunity to move forward, sharing and infusing knowledge and learning faster than we have ever done to adapt and reform our food systems using a multisectoral perspective. That is the task before us. I have yet to meet one person who disagrees.

